# Predicting phototherapy efficacy in vulvar lichen sclerosus using high-frequency ultrasound combined with superb microvascular imaging

**DOI:** 10.3389/fmed.2026.1756219

**Published:** 2026-04-16

**Authors:** Yifei Liu, Yu Lu, Yujuan Guo, Chuang Li, Kaikai Shen

**Affiliations:** Department of Ultrasound, Henan Provincial People’s Hospital, Zhengzhou, Henan, China

**Keywords:** microvessels, predictive modeling, ultrasonography, ultraviolet therapy, vulvar lichen sclerosus

## Abstract

**Objectives:**

To develop a non-invasive model predicting narrow-band UVB (NB-UVB) phototherapy efficacy in vulvar lichen sclerosus (VLS) using high-frequency ultrasound (HF-US, 24 MHz) and superb microvascular imaging (SMI) parameters.

**Methods:**

Forty-seven biopsy-confirmed VLS patients undergoing standardized NB-UVB (24 weeks) were enrolled (2021–2024). Baseline vascular density (VD), vessel index (VI, SMI), and dermal thickness (HF-US) were measured. SMI parameters were assessed in the most vascularized lesion area (comprehensive lesion coverage was performed). Clinical response (6 months post-treatment) followed a modified ISSVD composite endpoint. Univariate/multivariate logistic regression and ROC analyses (AUC, sensitivity, specificity) were used, adjusted for age and disease duration.

**Results:**

Responders (27/47, 57.4%) had significantly higher baseline VD (10.34 ± 2.01 vs. 8.37 ± 3.93, *P* = 0.030) and VI (7.33 ± 1.55 vs. 5.48 ± 2.53, *P* = 0.003). Baseline VD (OR = 1.85) and VI (OR = 2.02) were independent predictors. The combined model showed superior performance (AUC = 0.849, 88.9% sensitivity, 80.0% specificity).

**Conclusion:**

HF-US/SMI-derived baseline VD and VI enable accurate prediction of NB-UVB response in VLS, supporting individualized pre-treatment decisions.

## Introduction

Vulvar lichen sclerosis (VLS) is a chronic inflammatory dermatosis characterized by epidermal atrophy, progressive sclerosis, and debilitating symptoms including intractable pruritus, dyspareunia, and urinary dysfunction (1). Epidemiologically, VLS affects approximately 1.4% of the global female population, with incidence peaking in bimodal age groups (prepubertal children and postmenopausal women) (2). Population-based studies report a lifetime risk of 1:70 to 1:30, with significant underdiagnosis in resource-limited settings (3). The disease substantially impairs quality of life, with > 80% of patients reporting sexual dysfunction and 30–40% experiencing depressive symptoms (4). Most critically, VLS carries a 3–7% lifetime risk of vulvar squamous cell carcinoma, necessitating lifelong surveillance.

Current therapeutic guidelines recommend ultrapotent topical corticosteroids, such as clobetasol propionate 0.05%, as first line treatment, achieving initial symptom control in 70–90% of patients. However, relapse rates approach 50–80% within two years, requiring maintenance therapy that increases risks of skin atrophy and systemic absorption. Phototherapy has emerged as a steroid-sparing alternative, but the evidence base varies by wavelength. UVA1 phototherapy has stronger supporting literature for VLS, including an RCT comparing UVA1 with clobetasol for genital lesions (5) and reports of favorable response in refractory cases. In contrast, NB-UVB evidence for vulvar VLS is limited to case series and small cohort studies (6). We chose NB-UVB for this study due to its wider availability in clinical practice, lower equipment cost, and established safety profile for long-term use in dermatological conditions. Additionally, our institutional protocol prioritizes NB-UVB as a first-line phototherapeutic option for genital dermatoses based on local expertise and patient tolerance data. Systematic reviews of NB-UVB in VLS (limited to non-genital or mixed cohorts) demonstrate pooled response rates of 40–75%, with complete remission in 20–35% of cases (7). Nevertheless, 25–30% of patients derive minimal benefit, exposing them to unnecessary treatment costs and potential adverse effects like hyperpigmentation. This heterogeneity underscores the critical need for predictive biomarkers.

Conventional monitoring relies on subjective clinician assessment and patient reported outcomes, both vulnerable to recall and observer bias. Histopathological evaluation, while objective, is impractical for serial monitoring due to its invasiveness. High-frequency ultrasound (HF-US, 20–30 MHz) – the standard terminology for 24 MHz imaging (per WHO and ISUOG ultrasound nomenclature)—enables non-invasive quantification of dermal structural changes; specifically, reduced hyperechoic band thickness correlates with fibrosis improvement (8). However, it cannot assess microvascular dynamics essential for evaluating inflammatory activity. Conversely, superb microvascular imaging (SMI) detects low velocity blood flow (<1 cm/s) but lacks spatial resolution for epidermal quantification (9). This study pioneers the integration of SHF US (24 MHz) and SMI to simultaneously capture structural remodeling via dermal thickness measurement and microvascular activity through vessel density (VD) and vessel index (VI). This multimodal approach addresses fundamental limitations of single modality imaging by quantifying both the anti-fibrotic and immunomodulatory effects of phototherapy.

## Materials and methods

### Study design and participants

The prospectively study conducted at the Department of Ultrasound, Henan Provincial People’s Hospital between November 2021 and September 2024. Forty-seven female patients with pathologically confirmed VLS were enrolled.

Inclusion criteria:

(1) Patients with pathologically confirmed VLS [according to the International Society for the Study of Vulvovaginal Disease (ISSVD) 2015 diagnostic criteria] (8).
(2) Receiving narrow-band UVB phototherapy (initial dose of 0.1 J/cm^2^, twice weekly for 24 weeks).
(3) Not receiving topical hormone/immunomodulator therapy for ≥ 4 weeks.
(4) Voluntary acceptance of ultrasound examination.
(5) All patients participating in this study provided voluntary written informed consent after a comprehensive briefing.

Exclusion criteria:

(1) Pregnancy or lactation.
(2) Coexisting vulvar malignancies or other inflammatory/lichenoid dermatoses (e.g., lichen planus, psoriasis, lichen simplex chronicus, etc.), to avoid diagnostic confusion and potential interaction between disease processes.
(3) Received topical hormone/immunomodulator therapy within 4 weeks or had phototherapy contraindications (e.g., photosensitivity diseases).
(4) No tissue pathology results.

### Study registration and sample size calculation

The sample size calculation was based on a pilot study conducted at our institution (*n* = 15, independent from the current dataset, same inclusion/exclusion criteria and imaging protocol). The primary endpoint for calculation was the between-group difference in baseline VI (responders: 7.2 ± 1.8 vs. non-responders: 5.1 ± 2.3) from the pilot data. Using PASS 15.0 software, with a power (1-β) of 0.8, two-sided α of 0.05, and effect size (Cohen’s d) of 1.02, a minimum of 19 participants per group was required. Accounting for a 20% dropout rate, the final sample size was set at 47. Given the exploratory nature of the predictive model (2 primary predictors + 2 covariates), we followed the “10 events per predictor” rule (27 responders = 13.5 events per predictor), supporting adequate power for model development. External validation is planned in a multi-center cohort.

### Ultrasound imaging assessment

#### Equipment and parameters:

**FIGURE 1:**
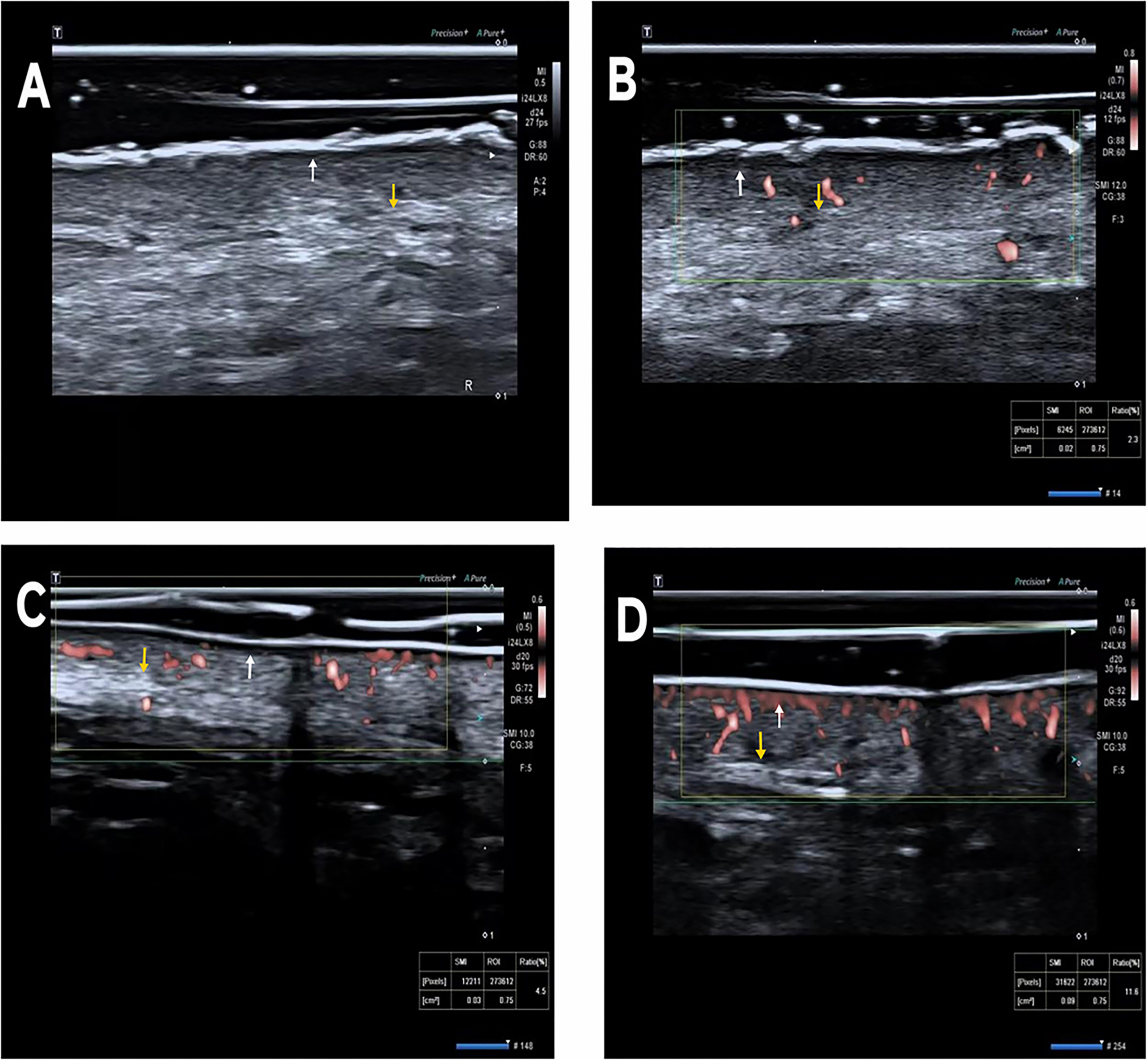
**(A)** Gray-scale ultrasound images of the control group. **(B)** VI measurement image of microvascular blood flow for the control group. **(C)** VLS group microvascular blood flow VI measurement image before treatment. **(D)** VLS group microvascular blood flow VI measurement image after treatment. The epidermis-dermis junction (marked with a white arrow) and the dermis-subcutaneous fat junction (marked with a yellow arrow) in the gray-scale ultrasound images.

Ultrasound equipment and probe used in the study. (A) Canon Aplio i900 ultrasound system; (B) PLI-2004BX linear probe (8–24 MHz) with pressure sensor. For dermal thickness assessment, we operated at 24 MHz to achieve axial resolution of 80 μm and lateral resolution of 160 μm - optimal for visualizing epidermal-dermal junctions (6). Probe pressure was standardized at < 0.2N using a pressure sensor to prevent tissue deformation (9) (see Figure 1).

All ultrasound examinations covered the entire lesion area to ensure comprehensive assessment of heterogeneous vascularization and structural changes. For SMI parameter measurement, the most vascularized region of interest (ROI) was selected because: (1) VLS lesions exhibit uneven pathological involvement, with preserved vascularized areas reflecting residual tissue responsiveness; (2) pilot data from our team showed that vascularized foci correlate with inflammatory activity, which is a key target of phototherapy; (3) non-vascularized fibrotic areas were also evaluated for dermal thickness but excluded from VD/VI analysis due to undetectable blood flow signals. This selection strategy ensures we capture the most biologically relevant region for predicting treatment response, as the presence of functional microvasculature may facilitate photonic energy absorption and immunomodulatory effects.

#### Measurement protocol

Dermal thickness: Defined as the maximal vertical distance from the epidermis-dermis interface to the dermal-subcutaneous fat junction in transverse scans. Three measurements were taken at the thickest portion of the lesion.

The hyperechoic epidermal layer and hypoechoic dermal layer were distinguished based on standard sonographic criteria (8, 9)

#### Microvascular parameters (SMI mode)

Vessel density (VD): Number of color-coded vessels per mm^2^ within a 5 × 5mm region of interest (ROI) centered on the most vascularized lesion area (10–12).

Vessel index (VI): Percentage area occupied by blood flow signals within the same ROI, calculated as (color pixel area / total ROI area) × 100% (13).

Ultrasound Data: To accurately capture the changes in ultrasound parameters, all SHF-US and SMI measurements (dermal thickness, VD and VI) were performed at two predefined time points: Baseline: Within 24 h prior to the initiation of the first NB-UVB phototherapy session. Post-treatment: Within 1 week after the completion of the final (24-week) NB-UVB phototherapy session. All follow-up measurements were performed by the same sonographers using the same equipment and standardized protocol as at baseline to maintain consistency.

### Quality control

#### Blinding procedure

To ensure the objectivity of outcome measurements, a blinding procedure was implemented. The image analysts (two sonographers with > 5 years of experience) were blinded to: (1) whether the images were acquired at baseline or post-treatment; and (2) the treatment response group of the patients from whom the images originated. All images were de-identified and randomized prior to analysis. The randomization process was conducted using a computer-generated random sequence (created with SPSS 27.0 software) to assign each image to a random position in the analysis queue. Specifically, all baseline and post-treatment images from all patients were pooled and sorted by a random number sequence, ensuring that the analysts could not discern the temporal order or group assignment of the images. The random sequence was generated by an independent statistician who was not involved in image acquisition or analysis, and the randomization list was stored securely without access by the analysts. The sonographers independently analyzed all images using machine-learning-assisted software, which automated the calculation of VD, VI, and dermal thickness to minimize manual intervention and potential bias during the analysis process.

Inter-observer concordance: Intraclass correlation coefficient (ICC) > 0.85 for all parameters.

Instrument calibration: Performed daily using tissue-mimicking phantoms.

### Narrowband UVB phototherapy protocol

#### Equipment:

Device: Waldmann UV7002K cabin (Herbert Waldmann GmbH & Co. KG).

Lamps: 48 TL-01 fluorescent tubes (311 nm peak wavelength).

Calibration: Monthly radiometry (Ocean Insight HDX-VIS-NIR spectrometer).

#### Dosimetry

1. Initial dose: Determined as 70% of the patient’s minimal erythema dose (MED), MED testing protocol: 8-dose geometric series (0.01–0.40 J/cm^2^) on non-lesional buttock skin.

2. Treatment schedule: Patients received twice-weekly treatments for 24 weeks.

The dose escalation protocol was as follows:

Weeks 1–2: Initial dose of 0.1 J/cm^2^ per session.

Weeks 3–12: Incremental increases of 0.02 J/cm^2^ per session.

Weeks 13–24: Maintenance at maximum dose of 0.5 J/cm^2^ (if tolerated).

#### Dose adjustment principles

No erythema: Increase by 0.02 J/cm^2^ at next session.

Grade 1 erythema (pink, non-painful): Maintain current dose.

Grade ≥ 2 erythema (red, painful, or with edema): Withhold treatment until resolution, then reduce dose by 0.02 J/cm^2^.

#### Treatment discontinuation criteria

Phototherapy was discontinued if any of the following occurred: (1) Grade 3 or higher acute phototoxicity reactions (as per CTCAE v5.0 criteria) (14), characterized by severe painful erythema with edema or blistering; (2) The patient experienced intolerable treatment-related adverse events and requested withdrawal; (3) The patient became pregnant; (4) Any contraindication to phototherapy (e.g., photosensitivity disorders, cutaneous malignancy) was diagnosed during the study period.

Session workflow:

Patients removed clothing below waist in private cubicle.

Technician positioned patient in standing posture with legs abducted.

Targeted irradiation of vulvar lesions using focused applicator.

Exposure duration calculated automatically (dose ÷ irradiance).

Post-treatment application of fragrance-free emollient.

### Efficacy assessment and grouping

#### Baseline data collection

Prior to the initiation of phototherapy, baseline data were systematically collected for all participants. This included: (1) Demographic data: age and body mass index (BMI); (2) Clinical characteristics: disease duration, history of prior topical corticosteroid use (12/47 patients had prior use > 4 weeks before enrollment, meeting inclusion criterion 3), and comorbidities (documenting the presence of autoimmune diseases potentially associated with VLS, such as autoimmune thyroid disease, alopecia areata, and vitiligo; as well as common systemic conditions like hypertension and type 2 diabetes mellitus); (3) Lesion characteristics: Lesion size (largest diameter in cm, with area calculated as π × (diameter/2)^2^ in cm^2^), the presence of multifocal involvement, and sclerosis severity were assessed by physical examination and standardized photography. Multifocal involvement was defined as the presence of two or more discrete, separate lesions with a distance of at least 1 cm between them; (4) Symptom severity: Intensity of pruritus and pain was evaluated using the Visual Analog Scale (VAS, 0–10) (15).

Baseline disease severity quantification:

ISSVD score components measured before phototherapy:

Baseline disease severity quantification:

The following components, as recommended by the International Society for the Study of Vulvovaginal Disease (ISSVD), were measured before phototherapy:

Symptom severity: Assessed using a VAS (0–10) for pruritus/pain, where 0 represents no symptoms and 10 represents the most severe, intolerable symptoms.

Objective signs:

Lesion area (cm^2^): Quantified via standardized photography with planimetric analysis.

Sclerosis severity: Clinically evaluated using a 4-point scale (16):

0: None (no induration);
1: Mild (slight induration with altered skin texture);
2: Moderate (marked induration, leathery feel);
3: Severe (extreme induration with scarring or architectural alteration).

Composite score: Calculated as VAS × 0.6 + Lesion area × 0.3 + Sclerosis score × 0.1.

### Clinical response assessment:

Clinical response was evaluated at 6 months post-treatment (i.e., immediately after completing 24 weeks of phototherapy) using a composite endpoint modified from International Society for the Study of Vulvovaginal Disease (ISSVD) terminology: ≥ 50% pruritus reduction + ≥ 30% lesion area reduction + ≥ 1-point reduction in sclerosis score (or ≥ 25% reduction from baseline, whichever is greater). Treatment response assessment was based on clinical symptoms (VAS for pruritus/pain), objective signs (lesion area via planimetry, sclerosis severity via clinical scoring), and sonographic changes; repeat biopsy was not performed due to ethical considerations of invasive procedures in responsive patients.

### Ethical approval

The study protocol was approved by the Institutional Review Board of Henan Provincial People’s Hospital (Approval No. 2025-06) in compliance with the Declaration of Helsinki.

### Informed consent process

#### Core discussion elements

Purpose: This study aims to develop an ultrasound-based method to predict phototherapy effectiveness for your vulvar condition.

Procedures: You will receive 24 weeks of NB-UVB therapy with pre/post ultrasound scans (SHF-US + SMI).

Risks: Phototherapy may cause temporary redness; ultrasound is non-invasive with no known risks.

Benefits: Personalized prediction may prevent ineffective treatment, but no direct therapeutic benefit.

Confidentiality: All data will be de-identified (ID codes replace names); images stored in password-encrypted PACS.

Voluntary Participation: You may withdraw anytime without affecting your standard care.

### Statistical analysis

Statistical analyses were performed using SPSS 27.0 software. Normality of continuous variables was tested using the Shapiro-Wilk test. Continuous variables following a normal distribution were presented as mean ± standard deviation and compared between responders and non-responders using independent samples *t*-tests. Non-normally distributed data were expressed as median (P25, P75) and compared using the Mann-Whitney U test. Categorical variables were summarized as frequencies (percentages) and compared using the Chi-square or Fisher’s exact test, as appropriate. To develop a predictive model for treatment response, univariate logistic regression was first conducted using exclusively baseline parameters. Variables with a *P* < 0.10 in the univariate analysis were subsequently entered into a multivariate logistic regression model to identify independent predictors, which was adjusted for the pre-specified clinical covariates of age and disease duration. The predictive performance of these independent baseline predictors, both individually and in combination, was assessed using Receiver Operating Characteristic (ROC) curve analysis, with the area under the curve (AUC) calculated and compared using the DeLong test. The robustness of the final multivariate model was evaluated by calculating the E-value to quantify the potential impact of unmeasured confounding. A two-sided *P* < 0.05 was considered statistically significant.

## Results

### Baseline characteristics of patients

A total of 47 female patients with pathologically confirmed VLS were enrolled and classified into non-responder (*n* = 20) and responder (*n* = 27) groups based on treatment efficacy evaluated at 6 months. Normality tests indicated that all continuous variables followed a normal distribution (*P* > 0.05). Independent samples *t*-tests demonstrated that responders had significantly higher baseline VD and VI compared to non-responders (*P* < 0.05). There were no significant differences between the two groups in terms of age, disease duration, or baseline dermal thickness (*P* > 0.05). Additionally, body mass index (BMI) was significantly lower in the responder group (*P* = 0.005). No significant differences were observed in the prevalence of comorbidities, lesion size, or multifocal involvement (*P* > 0.05). Twelve patients (25.5%) had a history of prior topical corticosteroid use > 4 weeks before enrollment. All participants were female and had no history of prior medication use; therefore, these variables were excluded from comparative analysis (Table 1).

**TABLE 1:** Baseline data of the two groups metric.

Metric	*N* = 47	No response (*n* = 20)	Response (*n* = 27)	t/Z/χ ^2^	*P*
Age, years [Mean ± SD]	45.98 ± 9.09	48.60 ± 9.76	44.04 ± 8.21	1.738	0.089
BMI, kg/m^2^ [Mean ± SD]	25.83 ± 2.36	26.99 ± 2.41	24.97 ± 2.34	2.987	0.005
Disease duration, years [Mean ± SD]	3.61 ± 1.76	3.90 ± 1.50	3.39 ± 1.94	0.974	0.335
Pre-treatment VD [Mean ± SD]	9.50 ± 3.11	8.37 ± 3.93	10.34 ± 2.01	−2.243	0.030
Pre-treatment VI [Mean ± SD]	6.54 ± 2.21	5.48 ± 2.53	7.33 ± 1.55	−3.106	0.003
Presence of Comorbidities, n (%)	18 (38.3%)	10 (50.0%)	8 (29.6%)	2.059	0.151
Lesion size, cm [Mean ± SD]	3.60 ± 1.32	3.95 ± 1.48	3.33 ± 1.13	1.621	0.112
Multifocal Lesions, n (%)	22 (46.8%)	12 (60.0%)	10 (37.0%)	2.303	0.129
ISSVD Composite Score, [Mean ± SD]	5.42 ± 1.28	5.58 ± 1.35	5.30 ± 1.22	0.754	0.455
Pre-treatment dermal thickness, mm [Mean ± SD]	1.48 ± 0.52	1.57 ± 0.56	1.41 ± 0.50	1.027	0.310
Prior topical corticosteroid use, n (%)	12 (25.5%)	5 (25.0%)	7 (25.9%)	0.012	0.913

### Changes in ultrasound parameters after treatment

Normality tests showed that all indicators after treatment conformed to normal distribution (*P* > 0.05). Independent sample *t*-tests were used to analyze differences in post-treatment VD, post-treatment VI, and post-treatment dermal thickness between the two groups. Results showed significant differences in these parameters between the groups (*P* < 0.05), with higher post-treatment VD and VI levels and lower post-treatment dermal thickness in the responder group (Table 2). Non-lesional vulvar skin (2 cm from the lesion edge) was measured as an internal control, with a mean thickness of 0.98 ± 0.21 mm (*n* = 20). Post-treatment dermal thickness in responders (0.79 ± 0.32 mm) was within the range of non-lesional skin (0.98 ± 0.21 mm), while non-responders (1.26 ± 0.55 mm) remained thicker than non-lesional controls, supporting fibrosis reversal rather than pathological atrophy. Supplementary Figure 1 shows the representative HF-US and SMI images of responders and non-responders before and after NB-UVB phototherapy, directly reflecting the differences in dermal thickness and microvascular activity changes between the two groups.

**TABLE 2:** Comparison of ultrasonic parameters between the two groups after treatment.

Metric	No response (*n* = 20)	Response (*n* = 27)	t/Z/χ ^2^	*P*
Post-treatment VD [Mean ± SD]	10.06 ± 4.39	13.66 ± 2.13	−3.730	0.001
Post-treatment VI [Mean ± SD]	7.48 ± 2.64	10.82 ± 1.98	−4.963	< 0001
post-treatment dermal thickness, mm [Mean ± SD]	1.26 ± 0.55	0.79 ± 0.32	3.671	0.001
Non-lesional dermal thickness (internal control), mm [Mean ± SD]	0.98 ± 0.21	0.96 ± 0.23	0.20	0.325

### Development of predictive model for phototherapy response

To construct a clinically applicable predictive model, we exclusively utilized parameters available prior to treatment initiation. Univariate logistic regression analysis identified three baseline factors significantly associated with treatment response: pre-treatment VD, pre-treatment VI, and BMI (all *P* < 0.05) (Table 3). These significant variables were subsequently incorporated into a multivariate logistic regression model, which was adjusted for age and disease duration. The multivariate analysis confirmed that both pre-treatment VD (OR = 1.85, 95% CI: 1.22–2.81, *P* = 0.004) and pre-treatment VI (OR = 2.02, 95% CI: 1.28–3.19, *P* = 0.002) were independent predictors of a favorable response to NB-UVB phototherapy (Table 4). Notably, BMI was no longer statistically significant in the multivariate model (*P* = 0.118), suggesting its effect was mediated through the microvascular parameters. The model demonstrated a good fit (Hosmer-Lemeshow test, *P* = 0.412).

**TABLE 3:** Univariate logistic regression analysis of baseline factors for predicting phototherapy response.

Metric	OR (95% CI)	SE	*z*	*P-*value
Pre-treatment VD	1.52 (1.08–2.14)	0.172	2.48	0.013
Pre-treatment VI	1.78 (1.21–2.62)	0.194	2.95	0.003
Pre-treatment dermal thickness (mm)	0.59 (0.23–1.52)	0.470	−1.09	0.274
BMI (kg/m^2^)	0.75 (0.59–0.95)	0.120	−2.38	0.017
Age (years)	0.95 (0.89–1.01)	0.031	−1.65	0.098
Disease duration (years)	0.86 (0.63–1.17)	0.152	−0.96	0.339
ISSVD composite score	0.85 (0.54–1.33)	0.227	−0.70	0.483

OR > 1 indicates a higher probability of response per unit increase in the metric. OR, Odds Ratio; CI, Confidence Interval; SE, Standard Error; VD, Vessel Density; VI, Vessel Index; BMI, Body Mass Index; ISSVD, International Society for the Study of Vulvovaginal Disease.

**TABLE 4:** Multivariate logistic regression model of baseline predictors for phototherapy response.

Metric	Adjusted OR (95% CI)	SE	*z*	*P-*value
Pre-treatment VD	1.85 (1.22–2.81)	0.209	2.91	0.004
Pre-treatment VI	2.02 (1.28–3.19)	0.231	3.11	0.002
BMI (kg/m^2^)	0.80 (0.61–1.05)	0.136	−1.56	0.118
Age (years)	0.96 (0.90–1.02)	0.032	−1.27	0.203

The model is adjusted for all variables listed in the table. OR > 1 indicates a higher probability of response per unit increase in the metric. OR, Odds Ratio; CI, Confidence Interval; SE, Standard Error; VD, Vessel Density; VI, Vessel Index; BMI, Body Mass Index.

### Predictive performance and validation of the baseline model

**FIGURE 2:**
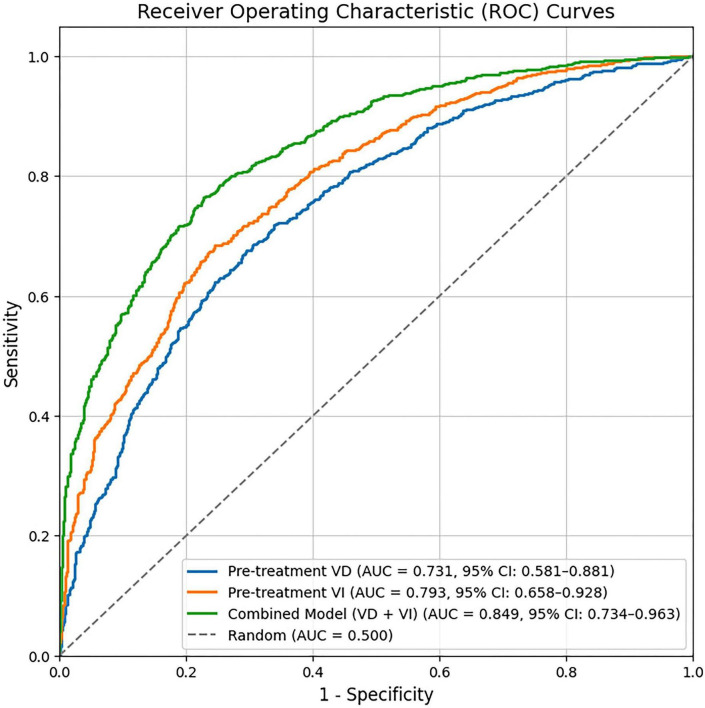
Receiver operating characteristic (ROC) curves for the baseline prediction model. The curves illustrate the predictive performance of pre-treatment vessel density (VD), pre-treatment vessel index (VI), and the combined model integrating both parameters for discriminating between phototherapy responders and non-responders. The combined model (solid blue line) demonstrates superior predictive accuracy compared to the single parameters.

The predictive performance of the individual baseline predictors identified in the multivariate analysis, as well as their combination, was evaluated using ROC curve analysis. As detailed in Table 5 and visualized in Figure 2, the pre-treatment VI demonstrated the highest discriminatory ability among the single parameters, with an AUC of 0.793 (95% CI: 0.658–0.928). The optimal cutoff value for pre-treatment VI was determined to be > 6.05, which yielded a sensitivity of 81.5% and a specificity of 75.0%. The pre-treatment VD also showed significant predictive value, with an AUC of 0.731 (95% CI: 0.581–0.881) and an optimal cutoff of > 8.85 (sensitivity 77.8%, specificity 70.0%). Crucially, the combined model, which integrated both pre-treatment VD and VI into a single predictive score, achieved superior performance compared to any single indicator. This combined model attained an AUC of 0.849 (95% CI: 0.734–0.963), with a sensitivity of 88.9% and a specificity of 80.0% at its optimal cutoff point (Figure 2). The Delong test confirmed that the AUC of the combined model was significantly larger than that of pre-treatment VD alone (*P* = 0.038) and showed a strong trend toward superiority over pre-treatment VI alone (*P* = 0.051).

**TABLE 5:** Predictive performance of baseline indicators and the combined model for phototherapy response.

Metric	AUC	95% CI for AUC	Sensitivity (%)	Specificity (%)	Youden index	Best cutoff value	*P*-value
Pre-treatment VD	0.731	0.581–0.881	77.8	70.0	0.478	> 8.85	0.004
Pre-treatment VI	0.793	0.658–0.928	81.5	75.0	0.565	> 6.05	< 0.001
Combined model (VD + VI)	0.849	0.734–0.963	88.9	80.0	0.689	–	< 0.001

The Combined Model is a predictive probability score derived from the multivariate logistic regression equation incorporating baseline VD and VI. AUC, Area Under the Curve; CI, Confidence Interval; VD, Vessel Density; VI, Vessel Index.

## Discussion

The typical pathologic changes of VLS are collagen fiber hyperplasia, fibrosis and chronic infiltration of inflammatory cells in the dermis (17). The thickness of the dermis represents the level of the tissue sclerosis and an important indicator of the degree of disease. Chen et al. (18) reported that high-frequency ultrasound were able to detect the hypoechoic dermal thickness in VLS lesions. It was also observed that the dermal thickness in the baseline of responder group was significantly lower than the one in the non-responder group after treatment, in consistent with those from the research by Ho et al. (19) who verified by histologic pathology that the extent of dermal fibrosis is negatively associated with phototherapy efficacy. It implies that phototherapy may counteract the dermal fibrosis by restricting the actions of fibroblasts and collagen deposition. It is worth mentioning that there is no statistical difference in the dermal thickness before treatment among the two groups (*P* > 0.05), indicating that the initial thickness of the dermis does not seem to be associated to the predictive factor in the efficacy of phototherapy, while the change in the thickness post treatment more effectively reflects the efficacy of treatment in comparison, which is in accord with the results of previous studies that phototherapy was hardly effective for advanced fibrotic lesions (20). Notably, post-treatment dermal thinning in responders was accompanied by improved sclerosis scores and symptom reduction, and thickness values were close to non-lesional skin, ruling out pathological atrophy. Measurement artifacts were minimized via standardized probe pressure and blinded analysis.

When operating in SMI mode, VD and VI can represent the blood perfusion status in the lesion field (11). In this study, we demonstrated that pre-treatment VD and VI values were significantly larger in the responder group than in the non-responder group (*P* < 0.05), indicating that a high baseline vascular activity may predispose the patients more sensitive to phototherapy. This is because phototherapy can cause injury of vascular endothelial cells, induce abnormal vessel closure, and regulate the local immune micro-environment (21). The VD and VI after the treatment were still elevated in the responder group (*P* < 0.05), which may be associated with stimulation of neovascularization as result of local inflammatory reaction, and the vascular parameters did not significantly improve in the non-responder group, suggesting that the vascular regulatory effects of phototherapy were not sufficient.

The results of the univariate logistic regression analysis, which exclusively utilized baseline parameters, confirmed that pre-treatment VD and pre-treatment VI were significantly associated with treatment response. Subsequent multivariate analysis, adjusted for key clinical confounders, established both pre-treatment VD and pre-treatment VI as independent predictors of a favorable outcome. The odds ratio for pre-treatment VD was 1.85, indicating that each unit increase in baseline VD was associated with an 85% higher odds of responding to phototherapy. Similarly, a unit increase in pre-treatment VI more than doubled the odds of response (OR = 2.02). This strong predictive value of baseline microvascular activity can be explained by the mechanism of phototherapy, where high baseline blood flow may facilitate the absorption and distribution of photonic energy, thereby enhancing its immunomodulatory effects on the dermal tissue (22). The fact that these parameters, measurable before treatment initiation, hold significant predictive power underscores their clinical utility for patient stratification. Furthermore, the profound reduction in dermal thickness observed in responders after treatment supports the established concept that phototherapy can reverse the fibrotic process in susceptible individuals (23).

In the ROC analysis of baseline parameters, pre-treatment VI demonstrated the highest discriminatory ability among single indicators (AUC = 0.793), with an optimal cutoff of > 6.05 yielding a sensitivity of 81.5% and specificity of 75.0%. It is important to note that the combined model, integrating the baseline VD and VI, achieved a superior predictive performance (AUC = 0.849), significantly outperforming either parameter alone. This underscores that a multi-parameter assessment of the pre-treatment microenvironment provides a more robust prediction than any single metric. Despite the modest sample size, this baseline model demonstrates considerable potential clinical utility.

Our findings affirm and significantly extend prior research on VLS imaging. While Chen et al. reported the value of SHF-US in detecting dermal thinning after treatment (AUC = 0.72) (18), their monomodal approach could not assess the vascular component. Crucially, our study establishes that baseline microvascular activity (VD and VI), which is undetectable by conventional Doppler, serves as an independent predictor of response. This is pathophysiologically plausible, as the efficacy of phototherapy is believed to depend on the induction of endothelial apoptosis and immunomodulation, processes that require pre-existing vascularity for targeted energy delivery (21). This mechanism also explains the poor response typically observed in advanced, fibrotic cases with low vascularity, a finding corroborated by Li HO’s histopathological data (19).

Contrary to Batchelor et al.’s assertion that vascular normalization signifies efficacy (24), we observed a sustained elevation of microvascular parameters in responders post-treatment. This suggests that the beneficial effect of phototherapy may be linked to an immunomodulation-driven neovascularization process [e.g., via VEGF upregulation (21)], rather than simple vascular quenching. This dichotomy underscores the dual role of phototherapy: simultaneously suppressing fibrotic processes while potentially amplifying localized immune activity in susceptible patients.

This study has several limitations. Its single-center design and relatively small sample size may limit the generalizability of the findings and introduce selection bias. Furthermore, we did not explore the dynamic effects of different phototherapy dosages or treatment durations on the ultrasound parameters. The absence of long-term follow-up data also prevents an assessment of the predictive value of these baseline parameters for disease recurrence. Although we adjusted for key clinical confounders and E-value analysis suggested that unmeasured confounders (e.g., genetic polymorphisms in VEGF pathways) would need strong associations to nullify our conclusions, their potential influence cannot be entirely ruled out. Future large-scale, multi-center prospective studies are warranted to validate our baseline predictive model and to explore the integration of molecular biomarkers (e.g., VEGF, TGF-β) with imaging features to build a more comprehensive predictive system for VLS.

## Conclusion

SHF-US combined with SMI provides a valuable, non-invasive tool for quantifying baseline microvascular and structural characteristics in VLS. Our study identifies pre-treatment VD and VI as robust, independent predictors of response to NB-UVB phototherapy. The novel predictive model integrating these baseline parameters demonstrates high accuracy and possesses the potential to serve as an objective clinical tool for screening patients likely to benefit from phototherapy, thereby facilitating personalized treatment decisions and avoiding ineffective therapeutic courses.

## Data Availability

The original contributions presented in the study are included in the article/Supplementary material, further inquiries can be directed to the corresponding authors.

## Supplementary material

Supplementary Figure 1 The representative HF-US and SMI images of responders and non-responders before and after NB-UVB phototherapy.
