# Supplementary figures and images for: Predicting phototherapy efficacy in vulvar lichen sclerosus using high-frequency ultrasound combined with superb microvascular imaging
Source: Front Med (Lausanne). 2026 Apr 16;13:1756219. doi: 10.3389/fmed.2026.1756219 (PMC13128633; doi:10.3389/fmed.2026.1756219)

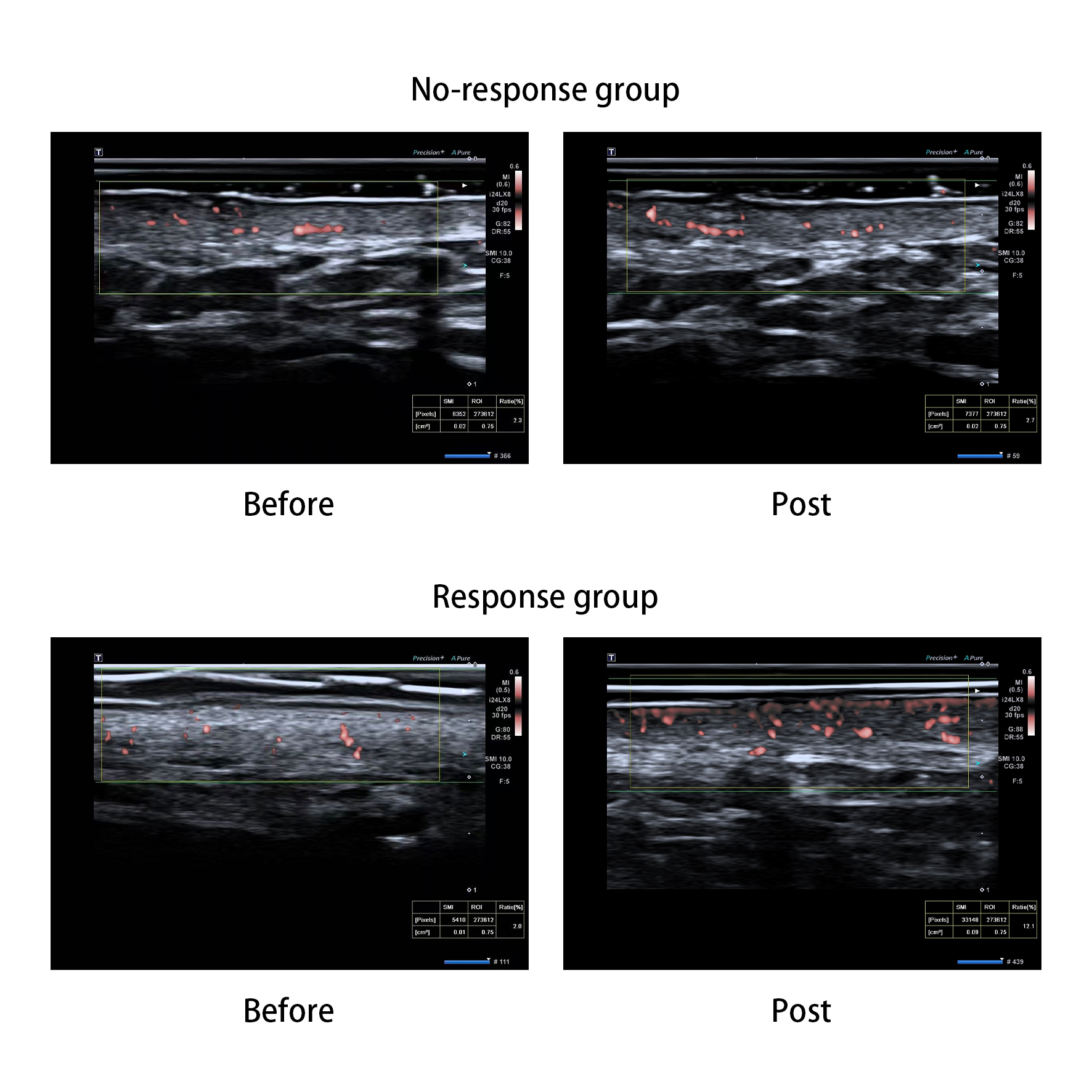

Supplement: Supplementary Figure 1 — The representative HF-US and SMI images of responders and non-responders before and after NB-UVB phototherapy. [file Image_1.jpeg]
